# Intermittent blood flow in the KHT sarcoma--flow cytometry studies using Hoechst 33342.

**DOI:** 10.1038/bjc.1990.259

**Published:** 1990-08

**Authors:** A. I. Minchinton, R. E. Durand, D. J. Chaplin

**Affiliations:** B.C. Cancer Research Centre, Vancouver, Canada.

## Abstract

The administration of the fluorescent DNA stain, Hoechst 33342, to mice bearing the KHT sarcoma, combined with flow cytometry, can be used to select cells according to their proximity to functional vasculature. Different protocols of administration of Hoechst 33342 were used in order to differentiate between the presence of temporary and chronically hypoxic cells. The results show a large difference in radiosensitivity between cells close to, and distant from, functional vasculature. However, this pattern of radiosensitivity is observed only when the staining period with Hoechst 33342 is short and coincides with the period of irradiation. When the radiation treatment is temporally divorced from the staining period then the radiosensitivity and staining intensity are not related. This result can be interpreted as indicating that hypoxic cells exist within this tumour as a result of fluctuations in tumour blood flow.


Br. J. Cancer (1990), 62, 195-200 © Macmillan Press Ltd., 1990

### Intermittent blood flow in the KHT sarcoma – flow cytometry studies using Hoechst 33342

A.I. Minchinton, R.E. Durand & D.J. Chaplin

*B.C. Cancer Research Centre, 601 West 10th Avenue, Vancouver, B.C., V5Z 1L3, Canada.*

**Summary** The administration of the fluorescent DNA stain, Hoechst 33342, to mice bearing the KHT sarcoma, combined with flow cytometry, can be used to select cells according to their proximity to functional vasculature. Different protocols of administration of Hoechst 33342 were used in order to differentiate between the presence of temporary and chronically hypoxic cells. The results show a large difference in radiosensitivity between cells close to, and distant from, functional vasculature. However, this pattern of radiosensitivity is observed only when the staining period with Hoechst 33342 is short and coincides with the period of irradiation. When the radiation treatment is temporally divorced from the staining period then the radiosensitivity and staining intensity are *not* related. This result can be interpreted as indicating that hypoxic cells exist within this tumour as a result of fluctuations in tumour blood flow.

There is unequivocal evidence for the presence of radiobiologically hypoxic cells in experimental tumours (Moulder & Rockwell, 1984; Vaupel, 1979) and sound clinical evidence that they play an important role in determining the sensitivity to radiotherapy of some human tumours (Bush *et al.*, 1978; Dische *et al.*, 1983; Henk, 1986, Overgaard *et al.*, 1986; Watson *et al.*, 1978). In addition, several studies indicate that sub-populations within tumours exhibit differential sensitivity to chemotherapeutic agents as a result of inherent sensitivity differences between aerobic and hypoxic cells (Teicher *et al.*, 1981) and drug penetration problems (Kerr & Kaye, 1987). There is little doubt therefore that the location of cells within tumours is an important determinant of their sensitivity to cancer treatment.

Tumour hypoxia may develop in at least two ways. The classical model of Thomlinson and Gray (1955) was based on histological evidence from human bronchial carcinoma, viable respiring cells occupying the space between the vascularized stroma and necrosis. Hypoxic cells were postulated to exist bordering necrotic areas at a relatively constant distance from blood vessels, the diffusion distance of oxygen in tissue. The Thomlinson and Gray model of 'diffusion limited' hypoxia greatly influenced radiobiological thinking for several decades and spawned many studies investigating ways in which the clinical radioresistance of the hypoxic cells could be overcome (for review see Fowler, 1983; Wardman, 1977).

The suggestion that hypoxic cells within tumours could result from local fluctuations in tumour blood perfusion made by Brown (1979) led to the recognition of a second type of hypoxia, now called acute, transient or 'perfusion limited' hypoxia. The simple model of cylindrical cords of tumour cells and a consistent gradient of oxygen away from the central blood vessel (Thomlinson & Gray, 1955) was therefore augmented with the presence of areas within the tumour where blood flow had transiently ceased.

A method developed at this laboratory has proved useful in examining the presence of acute hypoxia within experimental tumours. Chaplin *et al.* (1985, 1986, 1987) used the fluorescent DNA stain, Hoechst 33342, to stain cells around blood vessels. The stain is administered intravenously and cells close to functioning blood vessels are stained intensely whilst cells distant from the blood vessels receive little stain. This concentration gradient of the stain facilitates flow cytometric sorting of the cells after dissociation of the tumour (Olive *et al.*, 1985; Loeffler *et al.*, 1987). The sorted populations that result should be derived from different locations within the tumour relative to the blood supply at the time of staining. By assessing radiation cell survival of the different sorted fractions Chaplin *et al.* (1986, 1987) observed a dependency in response on the timing of the Hoechst 33342 administration relative to the radiation treatment. When the stain was administered during the irradiation period a large differential in radiosensitivity between the brightly and dimly stained cells was noted, but this disappeared when the stain was administered 20 minutes prior to the radiation treatment. From these experiments, it was concluded that the oxygen concentration, and hence radiosensitivity, was related to the fluorescent intensity when staining and irradiation were performed concurrently. When the staining and irradiation were divorced temporally, the opening and closing of blood vessels disrupted this pattern. This is probably because some bright cells residing close to blood vessels during the staining process subsequently became hypoxic due to local cessation of blood perfusion resulting in the radiosensitivity of the sorted population being decreased. Likewise, cells which are poorly stained because they reside close to a blood vessel of which flow had temporarily ceased during the staining period, would exhibit greater radiosensitivity than continuously hypoxic cells.

In the present study, several protocols of administration of Hoechst 33342 were used to investigate the presence of hypoxia in its different forms within the KHT sarcoma. Since there is presently great interest in the occurrence of hypoxia in tumours and particularly in the use of hypoxia activated cytotoxins (Chaplin, 1989), it is important to ascertain whether the presence of acute hypoxia is widespread in tumours of both animal models and humans.

### Materials and methods

### *Chemicals*

Hoechst 33342 (Sigma) was dissolved in phosphate buffered saline (PBS) at a concentration of 6.7 mmol $dm^{-3}$ (4 g $dm^{-3}$). At various times before irradiation, a volume of 0.1–0.2 $cm^3$ was intravenously administered by bolus injection or alternatively infused using an indwelling catheter in the tail vein of mice. A syringe pump (Model 2247, Harvard Apparatus, South Natick, Mass) fitted with a 'gastight' syringe (Hamilton, Reno, Nev.) was used to dispense the stain. In the tritiated thymidine incorporation experiments, 0.3 ml (0.3 mCi) (methyl-$^3$H)thymidine solution (74 GBq $mmol^{-1}$, Amersham) was used undiluted and administered i.p. 3 hours prior to Hoechst 33342 staining. Misonidazole, a gift from Dr C. Smithen, Roche Products, Welwyn, was administered i.p. 30 minutes prior to staining and irradiation at a dose level of 1 mmol $kg^{-1}$ (0.2 g $kg^{-1}$).

Correspondence: A.I. Minchinton, Department of Radiation Oncology, Division of Radiation Biology, Stanford University Medical Center - A058, Stanford, CA 94305-5105, USA.
Received 24 August 1989; and in revised form 23 March 1990.

*Tumour*

The KHT sarcoma described by Kallman *et al.* (1967) was grown subcutaneously on the sacral region of 8–12 week old C3H/He female mice (bred 'in house' or purchased from Charles River Inc., St Constant, Quebec) after inoculations of between $10^4$ and $10^5$ single cells in a volume of 0.01–0.05 $cm^3$ of PBS. Tumours of 400–600 mg excised weight were used unless stated otherwise.

*Procedure*

Most details of the procedures used have been reported previously (Chaplin *et al.*, 1986, 1987). Briefly, 5–15 minutes after the end of the irradiation period, the mice were killed, their tumours were excised, finely chopped using a scalpel, washed and centrifuged in PBS and then incubated at 37°C with trypsin (0.2%, Difco, Detroit, MI) and DNAse I (0.05%, Sigma) for 15–30 minutes. The suspensions were then washed and filtered through a 50 μm pore size polyester mesh. Specific numbers of cells were sorted and then plated using a soft agar clonogenic assay (Courtney, 1976). Mice were unanaesthetised, but restrained during irradiation, infusion and injection. Tumours were irradiated using 250 kVp X-rays filtered with 0.5 mm Cu at a dose rate of approximately 2.8 Gy $min^{-1}$.

*Fluorescent activated cell sorting*

Cells recovered from tumours were analysed and sorted using a Becton Dickinson FACS 440 with dual argon lasers operating at wavelengths of 350–360 nm (40 mW) and 488 nm (0.4 W). Forward light scatter (FLS) at 488 nm was used to 'gate out' small objects such as debris and erythrocytes and also large objects such as doublets and clumps of cells. Fluorescence intensity was measured perpendicularly at 449.5 ± 10 nm. The 488 nm laser was also used to measure peripheral light scatter (PLS); the amount of light scattered perpendicular to the incident beam. This parameter was used to estimate cell size. Dividing the fluorescent intensity by the PLS yields an estimate of cellular Hoechst 33342 concentration.

Sorting windows were based on Krough cylinders (Krough, 1919) assuming vessel diameter of 10 μm and tumour cord diameter of 290 μm. Figure 1 shows the two models which were used to subdivide the cell populations; 'equal depth' and 'equal volume'. Using the 'equal volume' model (left panel of Figure 1), the total number of cells obtained after dissociation of the tumour is divided into numerically equal fractions on the basis of fluorescence intensity or concentration of Hoechst 33342. The resultant 'shells' of such a hypothetical tumour cord become progressively narrower as the distance from the blood vessel increases. The 'equal depth' sorting model (right panel of Figure 1) subdivides the cells such that the width of each shell within the 'cord' is equal. As a consequence of this procedure the 'shell' closest to the blood vessel contains less than 1% of the total sorted cells, whilst the 'shell' most distant from the blood vessel contains about 20% of the sorted cells. If a small proportion of hypoxic cells is to be identified then 'equal volume' sorting should be more appropriate since the shell most distant from the vessel constitutes 10% of the tumour cells. Previous studies from this laboratory have used concentration of Hoechst 33342 as the basis of the sorting. In this study fluorescent intensity has been used.

DNA measurements were made using the one step method of Vindeløv (1977) with ethidium bromide. Approximately $10^4$ tumour cells from mice injected with tritiated thymidine were sorted directly into scintillation vials to which 5 $cm^3$ of Hydrofluor (National Diagnostics, Manville, NJ) was added and radioactive disintegrations were counted on a LKB 1214 scintillation counter (Turku, Finland).

### Results

The different ways in which cells derived from tumours can be sorted is illustrated in Figure 1. The effect these different techniques have on the DNA profiles of the various sorted fractions is shown in Figure 2. The manner in which Hoechst 33342 was administered in this particular case did not influence the resultant DNA profiles, i.e. profiles obtained from mice administered Hoechst 33342 as a 3 minute infusion are indistinguishable from those obtained when the stain was administered by i.v. infusion for 20–30 minutes. In Figure 2 the profile of each sort fraction shows several populations of cells with different DNA content. In the top left profile these have been labelled **a**, **b** and **c**. Population **a** represents host cells present within the tumour population. They are diploid in nature and separated from the main population **b**, which represent the tetraploid tumour cells. Population **c**, cells with greater than tetraploid DNA content, are those tumour cells in the S, $G_2$ and mitotic phases of the cell cycle. The proportion of diploid host cells within each sorted fraction does not exceed 10% of the total population and its proportion appears to increase slightly with increasing fraction number when sorting is based on concentration of Hoechst 33342, while it decreases when sorting is based on intensity. When cells were sorted on the basis of Hoechst 33342 derived fluorescent intensity an increase in the proportion of dividing cells (population **c**) with higher fraction number (greater fluorescent intensity) can be seen irrespective of whether 'volume' or 'depth' sorting geometry was implemented. However, this was not the case when cells were sorted on the basis of Hoechst 33342 concentration (i.e. dividing intensity by PLS). This finding combined with the fact that volume sorting is more rapid, led us to adopt a sorting technique based on 'volume' and fluorescence intensity for the radiobiological studies of hypoxia.

The dependence of proliferation state on the distance from blood vessels implied by these DNA histograms (when volume and fluorescence intensity is used as the basis of sorting) was confirmed using incorporation of tritiated thymidine into tumour cells from different sort fractions and is shown in Figure 3. Tritiated thymidine was administered 3½ hours prior to the Hoechst 33342 staining procedure. The staining procedure involved either a bolus injection or a 20 minute infusion of Hoechst 33342. Approximately five-fold greater incorporation was observed between the bright and dimly staining fractions, but we found no significant difference between the two staining techniques.

**Figure 1** Diagrammatic representation of theoretical tumour 'cord' based on the Krough (1919) cylinder. Cells can be sorted using the flow cytometer implementing 'equal depth' or 'equal volume' methodologies on the basis of Hoechst 33342 concentration or fluorescent intensity.

**Figure 2** DNA profiles of sorted cell populations using different sorting criteria as described in Figure 1. Fraction 1 represents the dimmest Hoechst 33342 derived fluorescent population whilst fraction 10 represents the brightest and therefore closest to functional vasculature. **a** represents the normal (host) diploid population. **b** represents the tumour (tetraploid) $G_0$ and $G_1$ populations while **c** represents the S, $G_2$ and M populations of tumour cells. Hoechst 33342 was administered to mice for a 3 minute infusion 10 minutes prior to sacrifice and tumour disaggregation. DNA profiles from mice infused Hoechst 33342 over an extended (20 minute) period showed similar patterns (now shown).

**Figure 3** Tritiated thymidine incorporation into DNA of cells from different sort fractions for injected. ○, *vs* infused, ●, tumour bearing mice. (Methyl-$^3$H)thymidine solution was administered to tumour bearing mice 3 hours prior to Hoechst 33342 bolus injection or 20 minute infusion. Sorting was based on 'equal volume' and fluorescent intensity. Error bars represent standard errors from 3 tumours.

With the establishment of a sorting technique which reliably produced populations from specific locations within the tumour relative to the vasculature, we then used several protocols of Hoechst 33342 administration to probe the nature of the radiobiological hypoxia in this tumour. First, a short infusion of stain was administered that coincided with the first 3 minutes of a 3.5 minute 10 Gy irradiation period (Figure 4). An order of magnitude difference in survival is observed between the dim and brightly staining cells, the brightly staining cells having a level of survival similar to that of fully oxygenated cells irradiated *in vitro*. If the animals were administered the hypoxic cell radiosensitizer misonidazole (1 mmol $kg^{-1}$, i.p.) 30 minutes prior to irradiation, the survival of all the fractions was reduced to that of the brightest cells. The survival of the unirradiated cells (the plating efficiency) for the different sort fractions is also shown and was independent of staining intensity at a level of about 20%. The 'all' fraction represents the mean response of an equal mixture of all the sorted cells.

When irradiation of the tumour was separated from the Hoechst 33342 staining period by 30 minutes, the resultant pattern of cell survival was typically that shown in Figure 5. Little difference in survival was seen between the different sort fractions, all having a surviving fraction approximating that of the entire population in the previous figure (when the staining and irradiation period were coincident). Figure 6 shows that when both the infusion of stain and irradiation periods were concurrent but prolonged over a 20 minute period the bright cells exhibited more radiosensitivity than the dim cells, but the differential in radiosensitivity was less than when the period of staining and irradiation were short as shown in Figure 4.

Initial sorting experiments assessing survival of different sort fractions exhibited considerable variability. Though more recently performed experiments show more uniformity in response (the figures show typical results), efforts were made to identify the source of the variability. Experimental factors such as the disaggregation process, the chopping of the tumour, the enzymatic incubations, were standardized but did not lead to decreased variability. The presence of a varying proportion of the central semiliquid 'pulp' found at the core of most KHT sarcomata, over about 300 mg in size, and its associated necrosis was thought to be a possible source of this variability. To test this possibility, tumour

**Figure 4** Clonogenic cell survival of sorted fractions after administration of Hoechst 33342 to tumour bearing mice by 3 minute infusion simultaneous with a 10 Gy irradiation period. Unirradiated (control plating efficiency), O, is compared to the effect of 10 Gy with, ▲, or without, ●, the pre-treatment with misonidazole (after correcting for unirradiated plating efficiency of each sorted fraction). Misonidazole was administered at a dose level of 1 mmol $kg^{-1}$, 30 minutes prior to radiation.

**Figure 5** Clonogenic cell survival of sorted fractions after administration of Hoechst 33342 to tumour bearing mice by bolus injection 30 minutes prior to 10 Gy radiation treatment. Irradiated cell survival is corrected for unirradiated plating efficiency.

**Figure 6** Clonogenic cell survival of sorted fractions after administration of Hoechst 33342 to tumour bearing mice for 20 min infusion simultaneous with a 20 minute 10 Gy irradiation period. Unirradiated (control plating efficiency) is compared to the effect of 10 Gy. Irradiated cell survival is corrected for unirradiated plating efficiency.

tissue was separated into two parts by gently rinsing the 'pulp' away from the well structured part of the tumour and examining the cell survival after radiation of cells derived from both parts after sorting. The irradiation and staining procedure was as described in Figure 4, short and simultaneous. Figure 7 shows that the plating efficiency as well as the radiosensitivity of the different sort fractions appear identical for both parts of the tumour.

Tumour size was also investigated as a possible source of variability. Chaplin *et al.* (1986) observed that SCCVII tumours smaller that 350 mg showed a differential in radiosensitivity when the stain was injected 20 minutes prior to the radiation treatment; suggesting that acute hypoxia developed in tumours greater than that size. Our studies with the KHT sarcoma are shown in Figure 8. In each case the Hoechst 33342 was administered intravenously for the first 3 minutes of a 3.5 minute irradiation period similar to the protocol used to obtain the results in Figure 4. Two sizes of tumours were selected and the radiation response of the sorted fractions was assessed. Panel a shows the survival of the different fractions of cells sorted from six tumours ranging between 170 and 290 mg. Panel b show the sort fractions of cells from five larger tumours of excised weight between 718 and 913 mg. Both panels indicate that the tumours contain both oxygenated and hypoxic cells. The larger tumours show both a smaller differential between the bright and dimly fluorescent cells and greater variability than the medium sized tumours.

### Discussion

The diffusion of oxygen from the vasculature to the cells of the tumour has been modelled in several ways. Krough (1919) described the concept of cords of muscle cells supplied with nutrients and oxygen from a central blood vessel. Thomlinson and Gray (1955) described a similar histological architecture in tumours, but also described tumour cords surrounded by a vascularised stroma with necrosis present at the centre. These two models of 'outward' and 'inward' diffusion are analogous to contemporary models employed

**Figure 7** Clonogenic cell survival of sorted fractions, from two regions of tumours, following staining and irradiation as in Figure 4. The semi-liquid pulp region (triangles), is compared with the well structured region (circles), of the tumour. Cell survival after 10 Gy (closed symbols) is corrected for unirradiated plating efficiency (open symbols).

**Figure 8** The effect of tumour size on the clonogenic cell survival of sorted fractions. The other details are the same as in Figure 4. **a** shows the mean ( ± s.e.) of survival for the different sort fractions in six medium sized tumours of 173, 219, 219, 250, 272 and 291 mg excised weight. **b** shows similar data from five large tumours of 718, 734, 787, 805 and 913 mg.

when sorting cells from tumours and spheroids respectively (Durand *et al.*, 1990).

In previous studies performed at this laboratory, using the SCCVII tumour, the sorting was performed on the basis of Hoechst 33342 concentration, that is, dividing the fluorescence intensity by the peripheral light scatter (PLS). In this study only fluorescence intensity was used. We chose to do this because unpublished studies performed using concentration as the basis of the sorting often failed to result in a difference of radiosensitivity between the bright and dim cells in the KHT sarcoma, even when the Hoechst 33342 was administered concomitant with the irradiation period. In addition, the results shown in Figure 2 suggest that sorting on the basis of concentration did not resolve populations of cells that originated from different locations within the tumour relative to the vasculature. The reasons why the sorting method used previously and effectively in the SCCVII tumour failed to resolve similar populations from the KHT that were proportional to vascular proximity may lie in the differences between the two tumours. Although both tumours are extremely poorly differentiated and have comparable hypoxic fractions, the KHT unlike the SCCVII is extensively necrotic. We believe this factor and its contribution to the heterogeneity of the KHT sarcoma may result in a cellular staining patterns that only partially reflect the proximity of vasculature. Despite this heterogeneity the data shown in Figure 2 provide good evidence that sorting tumour cells on the basis of intensity of Hoechst 33342 was effective in selecting cells as a function of distance from the vasculature. Further evidence of the suitability of this sorting technique was obtained from the results in Figure 3 indicating an increased uptake of tritiated thymidine in brightly fluorescent cells. It is interesting to note that the profiles shown in Figures 2 and 3 can result from either a 3 minute infusion of Hoechst 33342 or a prolonged infusion of 20–30 minutes. The radiobiological response of the fractions, however, is very dependent on the timing of the staining relative to the irradiation as is shown in Figures 4 and 5. This is probably because the fraction of cells that is transiently hypoxic at any moment in time is relatively small and, although it can profoundly alter cell survival of a sorted population, it would not be expected to alter appreciably the overall DNA profiles shown in Figure 2 or the proportion of cells incorporating tritiated thymidine shown in Figure 3.

To probe the nature of hypoxia within the tumour, three methods of stain administration were employed. Figure 4 shows that when the stain and irradiation were carried out rapidly and simultaneously a large differential in cell survival was observed between the bright and dim populations. This differential disappeared either when misonidazole, a hypoxic cell radiosensitizer of hypoxic cells was administered, or when the stain and irradiation was separated by 20–30 minutes as illustrated in Figure 5. The differential however was partially restored when both the staining and irradiation was prolonged over a 20 minute period as shown in Figure 6. We interpret these results as indicating that the blood flow to at least some regions of the tumour is intermittent resulting in a breakdown in the relationship between sort fraction and oxygen status. This conclusion is in contrast to that made by Siemann and Keng (1988) who suggested on the basis of experiments using the injection of stain 20 minutes prior to irradiation, that chronic hypoxia was the dominant form of hypoxia within the KHT/Ro sarcoma. Several explanations for these divergent conclusions can be suggested. Siemann and Keng (1988) used a different variant of the tumour used in this study and implanted it intramuscularly rather than subcutaneously. Although a comparison between the two sites has not been made for either of these versions of the KHT tumour, Brown (1979) documented differences in the radiosensitivity of the RIF-1 tumour when implanted in these two sites. Therefore, it is possible that histological differences both inherent within the tumour and related to the site of implantation, may contribute to the differences observed by Siemann and Keng (1988) and those found in this study. However, we feel it is important when assessing the presence of acute hypoxia to use two different staining protocols, one where the stain is administered simultaneously with the radiation treatment and another where the staining is carried out temporally divorced from the radiation period. By comparing the pattern of radiosensitivity of the different sort fractions using two protocols, definitive evidence for the presence of acute or transient hypoxia can be obtained.

Our results further suggest that the presence of the semi-liquid pulp found in large KHT tumours did not appear to influence the response of the sorted population. This is surprising considering the grossly disparate appearance of the different regions of the tumour and the extensive necrosis evident microscopically as debris within the semi-liquid 'pulp' part of the tumour. Urtasun (1972) and Jirtle (1978) both observed viable cells within the grossly necrotic regions of tumours and our observation supports theirs and further suggests that there is no inherent difference in the radiosensitivity of the viable cells derived from these different regions.

The size of the tumour was more important however. Large tumours between 700 and 900 mg showed less of a differential in radiosensitivity than tumours between 170 and 290 mg. It is clear therefore that tumour size, as found in the SCCVII tumour (Chaplin *et al.*, 1986), is an important determinant of response in this system. Acute hypoxia is certainly demonstrable in medium sized KHT tumours, but the survival data exhibit more variability than was seen in the SCCVII tumour and it is therefore not possible, from these data, to correlate the appearance of acute hypoxia with a particular tumour size as was possible from the study of Chaplin *et al.* (1986). It is conceivable that in large tumours, the variability in survival of the different sorted fractions, may mask the ability of this technique to confirm the presence of acute hypoxia.

In conclusion, our data support the findings of Tannock (1968) who demonstrated a relationship between proliferation status of cells and proximity to the blood supply. Our studies suggest the blood supply in the KHT sarcoma is unstable and results in areas of temporary hypoxia. These hypoxic cells will, at some time, become reoxygenated, but if these acutely hypoxic cells are present in human tumours their existence during radiotherapy could represent a population of resistant cells of considerable clinical importance. The mechanism underlying this unstable blood flow is not well understood but may be related to interstitial pressure, blockage of vessels by normal or tumour cells and/or other factors.

Techniques that assess the potency of radiation or other cytotoxic agents to kill tumour cells cannot usually distinguish the relative effectiveness of these treatments on different regions or sub-populations within the tumour. As a result, agents that are very effective at killing specific regions or sub-populations within tumours are not easily studied since their overall potency is masked by regions or populations of cells that are not equally affected. The methodology used in this and previous similar studies may be an important tool in the identification and application of potential therapeutic agents.

The authors acknowledge the technical assistance of William Grulkey, Nancy LePard and Denise McDougal. This work is supported by grant 40459 from the NCI (USA) and by the Medical Research Council of Canada.

### References

BROWN, J.M. (1979). Evidence for acute hypoxic cells in mouse tumours and a possible mechanism of reoxygenation. *Br. J. Radiol.*, **52**, 650.

BUSH, R.S., JENKIN, R.D.T., ALLT, W.E.C. & 4 others (1978). Definitive evidence for hypoxic cells influencing cure in cancer therapy. *Br. J. Cancer*, **37** (suppl. 37), 302.

CHAPLIN, D.J., DURAND, R.E. & OLIVE, P.L. (1985). Cell selection from a murine tumour using the fluorescent probe Hoechst 33342. *Br. J. Cancer*, **51**, 569.

CHAPLIN, D.J., DURAND, R.E. & OLIVE, P.L. (1986). Acute hypoxia in tumours: implications for modifiers of radiation effects. *Int. J. Radiat. Oncol. Biol. Phys.*, **12**, 1279.

CHAPLIN, D.J., OLIVE, P.L. & DURAND, R.E. (1987). Intermittent blood flow in murine tumours: Radiobiological effects. *Cancer Res.*, **47**, 597.

CHAPLIN, D.J. (1989). Hydralazine-induced tumor hypoxia: A potential target for cancer chemotherapy. *J. Natl Cancer Inst.*, **81**, 618.

COURTNEY, V.D. (1976). A soft agar colony assay for lewis lung tumour and $B_{16}$ melanoma taken directly from the mouse. *Br. J. Cancer*, **34**, 39.

DISCHE, S., ANDERSON, P.J., SEALY, R. & WATSON, E.R. (1983). Carcinoma of the cervix- anaemia, radiotherapy and hyperbaric oxygen. *Br. J. Radiol.*, **56**, 251.

DURAND, R.E., CHAPLIN, D.J. & OLIVE, P.L. (1990). Cell sorting with Hoechst and Carbocyanine dyes as perfusion probes in spheroids and tumours. In *Methods in Cell Biology – Volume 23: Flow Cytometry*, Crissman, H.A. & Darzynkiewicz, Z. (eds). Academic Press: Orlando, FL.

FOWLER, J.F. (1983). La Ronde–radiation sciences and medical radiology. *Radiother. Oncol.*, **1**, 1.

HENK, J.M. (1986). Late results of a trial of hyperbaric oxygen and radiotherapy in head and neck cancer: A rationale for hypoxic cell sensitizers? *Int. J. Radiat. Oncol. Biol. Phys.*, **12**, 1339.

JIRTLE, R. & CLIFTON, K.H. (1978). The effect of tumour size and host anemia on tumor cell survival after irradiation. *Int. J. Radiat. Oncol. Biol. Phys.*, **4**, 395.

KALLMAN, R.F., SILINI, J. & VAN PUTTEN, L.J. (1967). Factors influencing the quantitation of the *in vivo* survival of cells from solid tumours. *J. Natl Cancer Inst.*, **39**, 539.

KERR, D.J. & KAYE, S.B. (1987). Aspects of cytotoxic drug penetration with particular reference to anthracyclines. *Cancer Chemother. Pharmacol.*, **19**, 1.

KROUGH, A. (1919). The number and distribution of capillaries in muscle with the calculation of the oxygen pressure head necessary for supplying the tissue. *J. Physiol.*, **52**, 409.

LOEFFLER, D.A., KENG, P.C., WILSON, K.M. & LORD, E.M. (1987). Comparison of fluorescence intensity of Hoechst 33342 – stained EMT6 tumour cells and tumour-infiltrating host cells. *Br. J. Cancer*, **56**, 571.

MOULDER, J.E. & ROCKWELL, S. (1984). Hypoxic fractions of solid tumours: experimental techniques, methods of analysis, and a survey of existing data. *Int. J. Radiat. Oncol. Biol. Phys.*, **10**, 695.

OLIVE, P.L., CHAPLIN, D.J. & DURAND, R.E. (1985). Pharmacokinetics, binding and distribution of Hoechst 33342 in spheroids and murine tumours. *Br. J. Cancer*, **52**, 739.

OVERGAARD, J., SAND HANSEN, H., JORGENSEN, K. & HJELM, M. (1986). Primary radiotherapy of larynx and pharynx carcinoma–an analysis of some factors influencing local control and survival. *Int. J. Radiat. Oncol. Biol. Phys.*, **12**, 515.

SIEMANN, D.W., & KENG, P.C. (1988). Characterization of the radiation resistant hypoxic cell subpopulation in KHT sarcomas. (ii) Cell sorting. *Br. J. Cancer*, **58**, 296.

TANNOCK, I.F. (1968). The relation between cell proliferation and vascular system in a transplanted mouse mammary tumour. *Br. J. Cancer*, **22**, 258.

THOMLINSON, R.H. & GRAY, L.H. (1955). The histological structure of some human lung cancers and the possible implications for radiotherapy. *Br. J. Cancer*, **9**, 539.

TEICHER, B.A., LAZO, J.S. & SARTORELLI, A.C. (1981). Classification of antineoplastic agents by their selective toxicities toward oxygenated and hypoxic tumour cells. *Cancer Res.*, **41**, 73.

URTASUN, R.C. & MERZ, T. (1972). *In vivo* studies of X-irradiation damage and repair in mammalian tumour cells. *Radiology*, **102**, 707.

VAUPEL, P. (1979). Oxygen supply to malignant tumours. In *Tumour Blood Circulation: Angiogenesis, Vascular Morphology and Blood Flow in Experimental and Human Tumours*, Peterson, H.-I. (ed) p. 143. CRC Press: Boca Raton, FL.

VINDELØV, L.L. (1977). Flow microfluorometric analysis of nuclear DNA in cell suspensions from solid tumours and cell suspensions. *Virchows Arch. B. Cell Path.*, **24**, 227.

WARDMAN, P. (1977). The use of nitroaromatic compounds as hypoxic cell radiosensitizers. *Curr. Topics Radiat. Res. Q.*, **11**, 349.

WATSON, E.R., HALNAN, K.E., DISCHE, S. & 3 others (1978). Hyperbaric oxygen and radiotherapy. A Medical Research Council trial in carcinoma of the cervix. *Br. J. Radiol.*, **51**, 879.


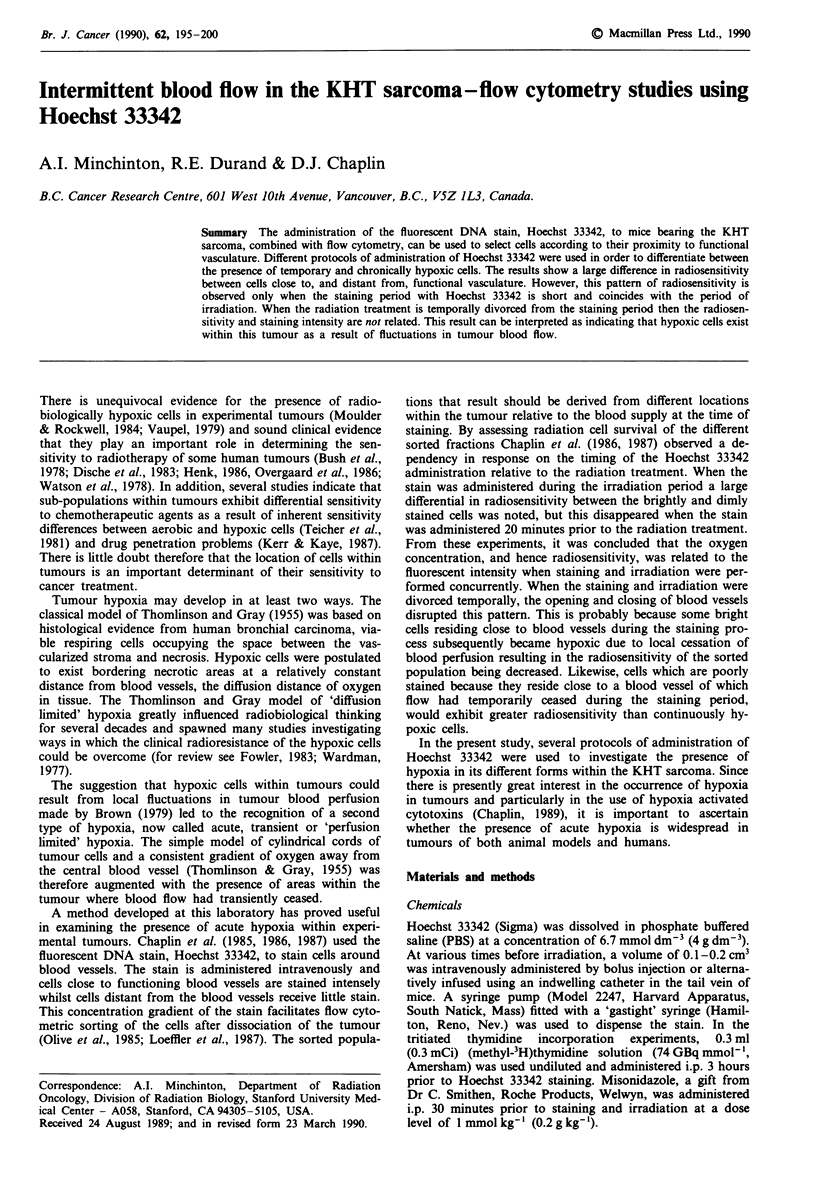

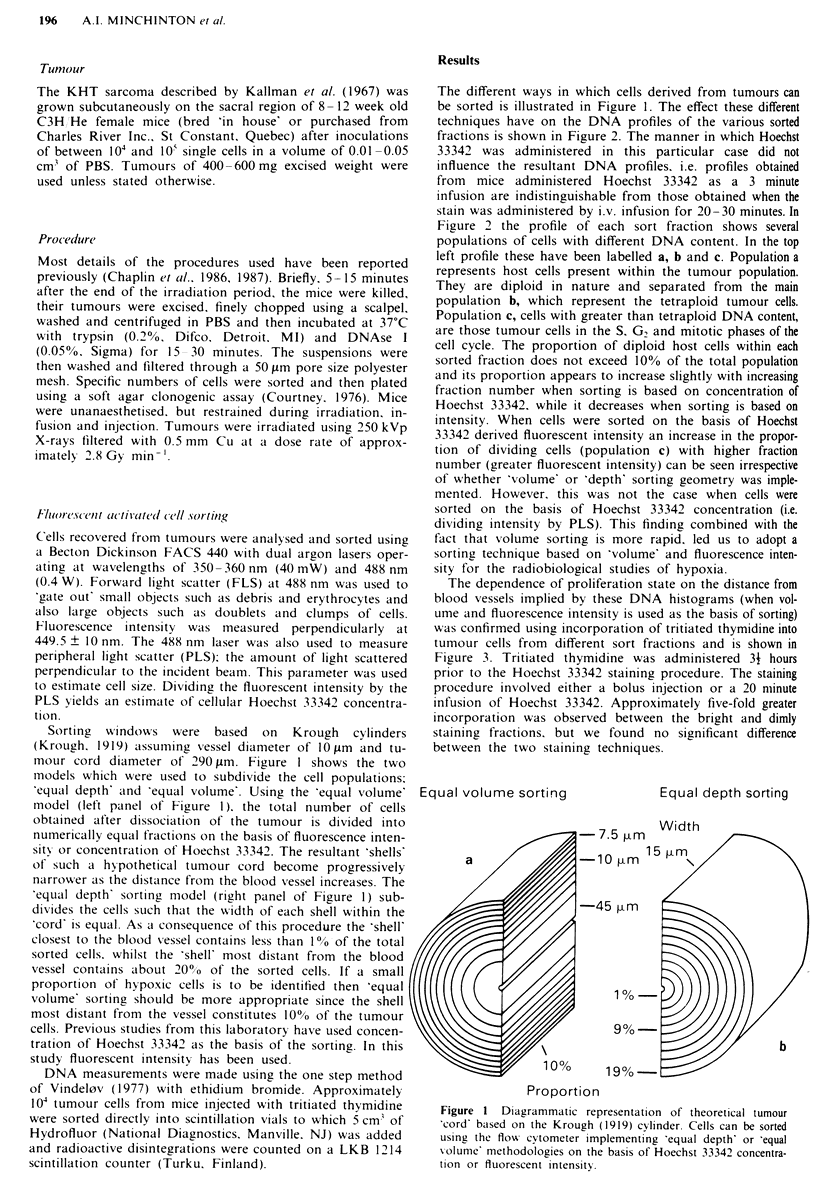

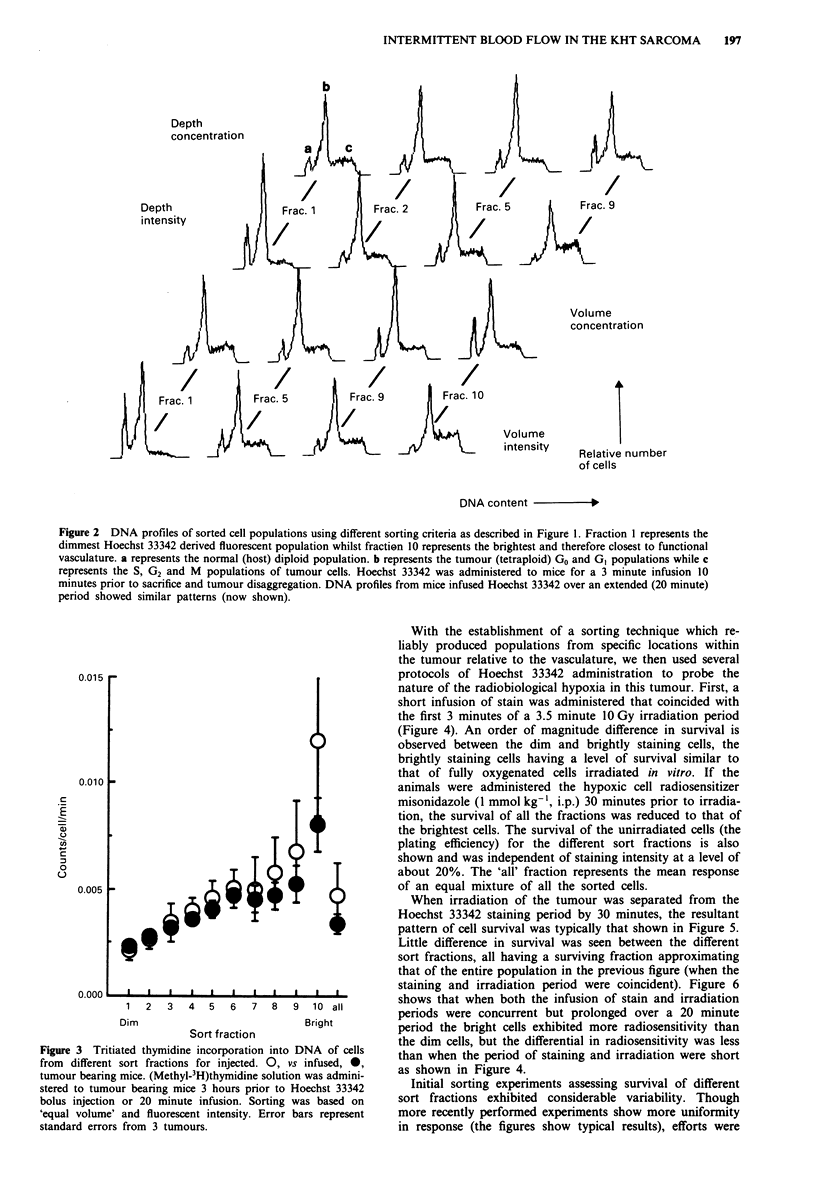

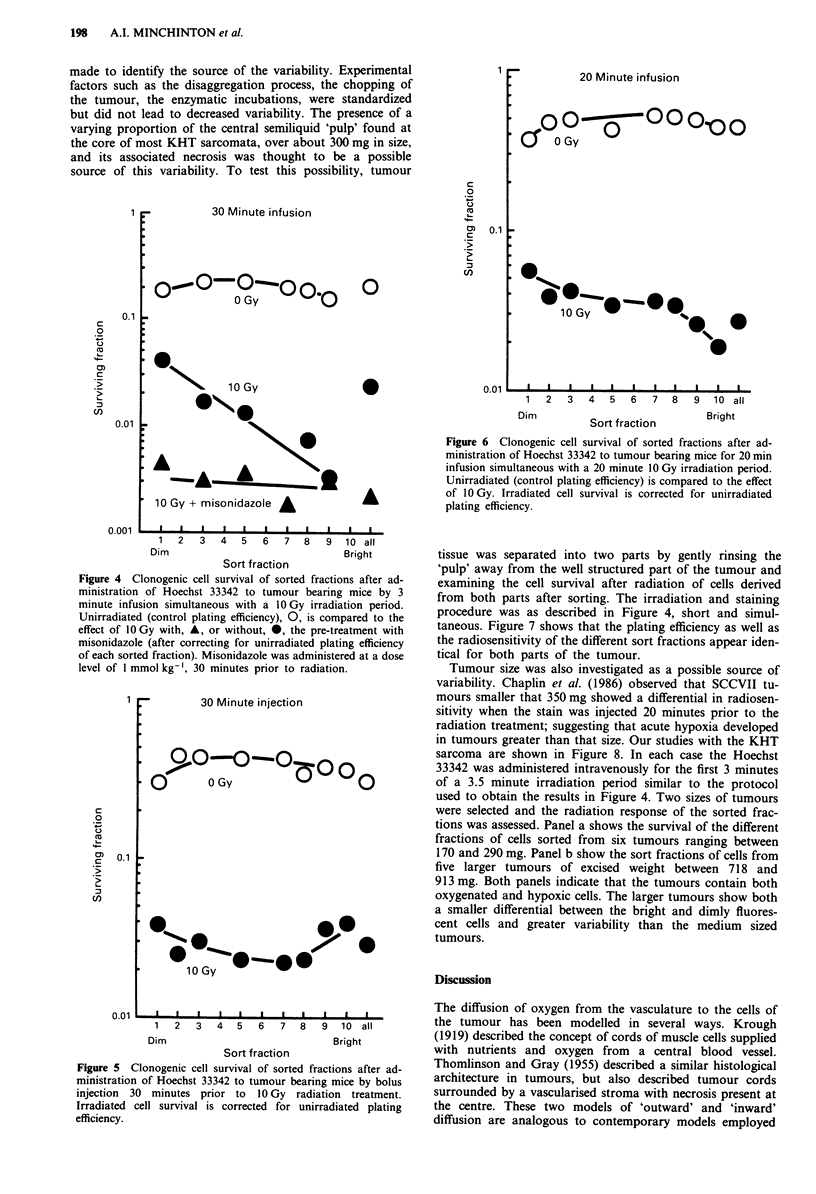

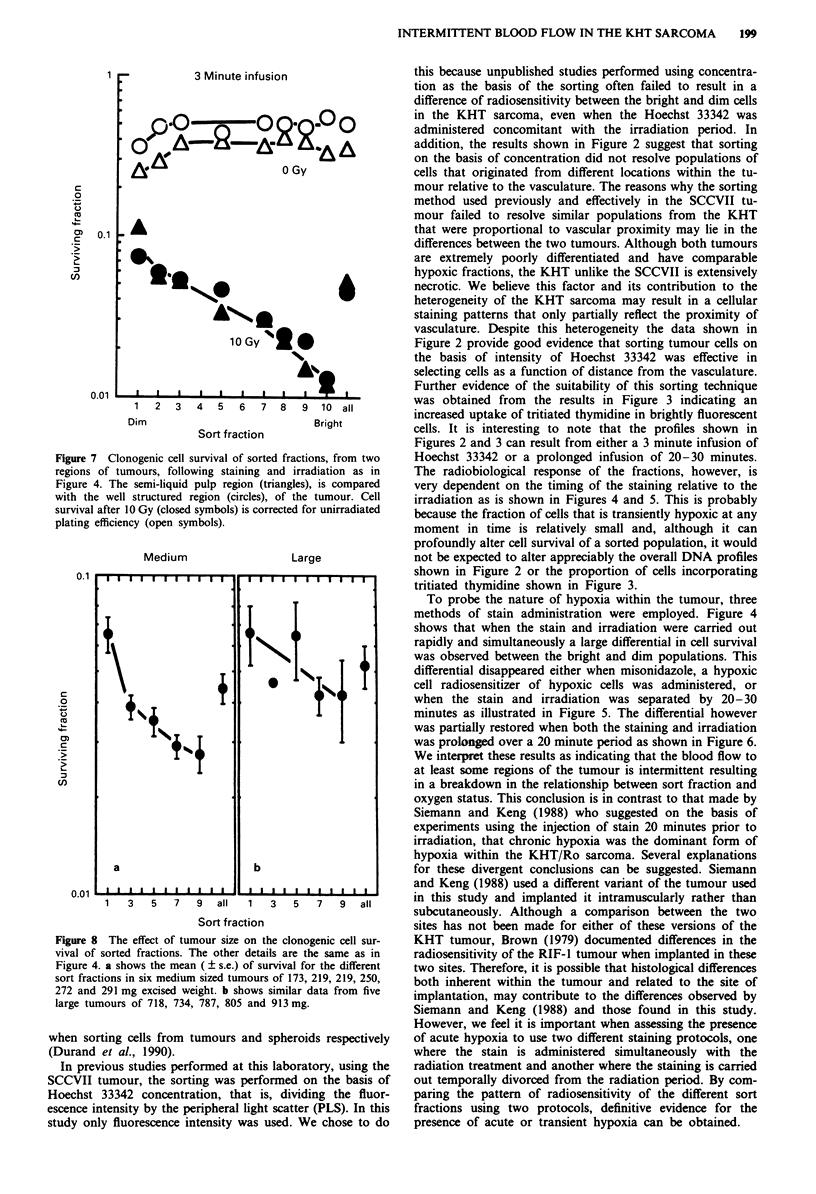

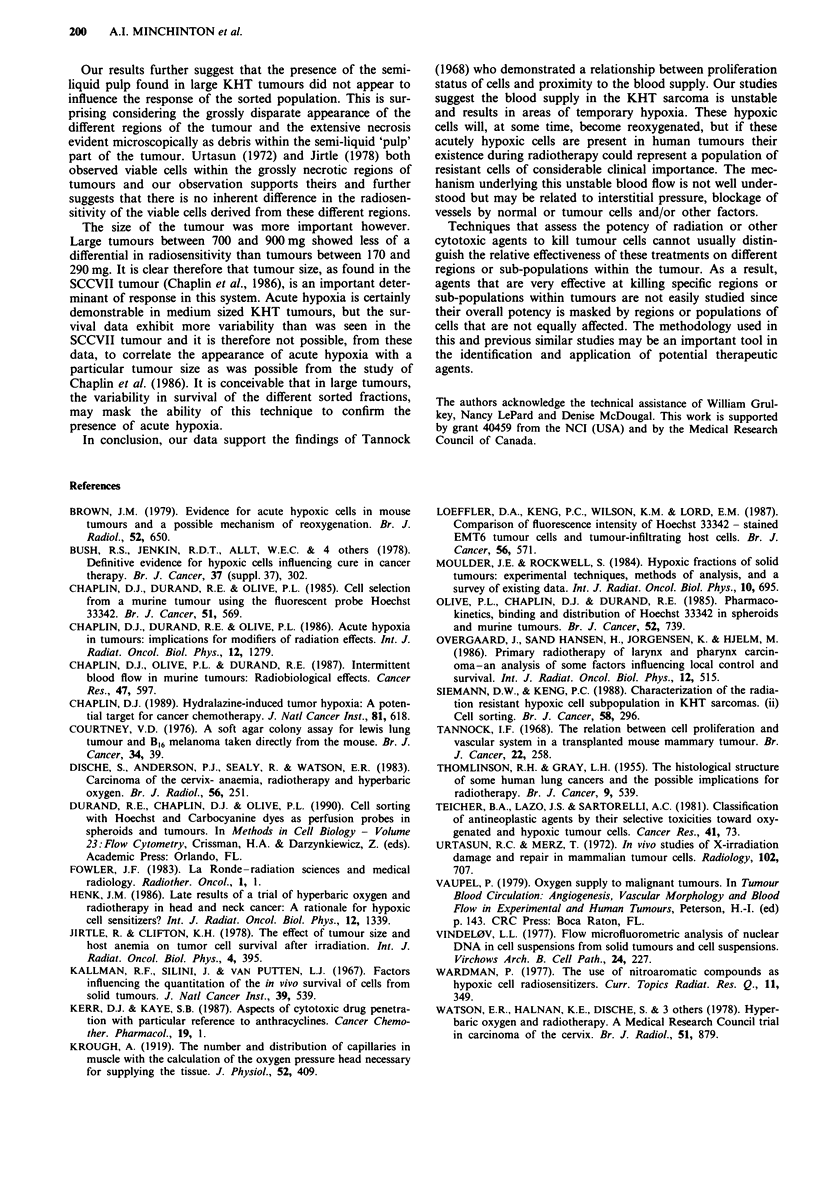


## References

[OCR_00842] Brown J. M. (1979). Evidence for acutely hypoxic cells in mouse tumours, and a possible mechanism of reoxygenation.. Br J Radiol.

[OCR_00847] Bush R. S., Jenkin R. D., Allt W. E., Beale F. A., Bean H., Dembo A. J., Pringle J. F. (1978). Definitive evidence for hypoxic cells influencing cure in cancer therapy.. Br J Cancer Suppl.

[OCR_00857] Chaplin D. J., Durand R. E., Olive P. L. (1986). Acute hypoxia in tumors: implications for modifiers of radiation effects.. Int J Radiat Oncol Biol Phys.

[OCR_00852] Chaplin D. J., Durand R. E., Olive P. L. (1985). Cell selection from a murine tumour using the fluorescent probe Hoechst 33342.. Br J Cancer.

[OCR_00867] Chaplin D. J. (1989). Hydralazine-induced tumor hypoxia: a potential target for cancer chemotherapy.. J Natl Cancer Inst.

[OCR_00862] Chaplin D. J., Olive P. L., Durand R. E. (1987). Intermittent blood flow in a murine tumor: radiobiological effects.. Cancer Res.

[OCR_00870] Courtenay V. D. (1976). A soft agar colony assay for Lewis lung tumour and B16 melanoma taken directly from the mouse.. Br J Cancer.

[OCR_00875] Dische S., Anderson P. J., Sealy R., Watson E. R. (1983). Carcinoma of the cervix--anaemia, radiotherapy and hyperbaric oxygen.. Br J Radiol.

[OCR_00887] Fowler J. F. (1983). The second Klaas Breur memorial lecture. La Ronde--radiation sciences and medical radiology.. Radiother Oncol.

[OCR_00891] Henk J. M. (1986). Late results of a trial of hyperbaric oxygen and radiotherapy in head and neck cancer: a rationale for hypoxic cell sensitizers?. Int J Radiat Oncol Biol Phys.

[OCR_00896] Jirtle R., Clifton K. H. (1978). The effect of tumor size and host anemia on tumor cell survival after irradiation.. Int J Radiat Oncol Biol Phys.

[OCR_00901] Kallman R. F., Silini G., Van Putten L. M. (1967). Factors influencing the quantitative estimation of the in vivo survival of cells from solid tumors.. J Natl Cancer Inst.

[OCR_00906] Kerr D. J., Kaye S. B. (1987). Aspects of cytotoxic drug penetration, with particular reference to anthracyclines.. Cancer Chemother Pharmacol.

[OCR_00911] Krogh A. (1919). The number and distribution of capillaries in muscles with calculations of the oxygen pressure head necessary for supplying the tissue.. J Physiol.

[OCR_00916] Loeffler D. A., Keng P. C., Wilson K. M., Lord E. M. (1987). Comparison of fluorescence intensity of Hoechst 33342-stained EMT6 tumour cells and tumour-infiltrating host cells.. Br J Cancer.

[OCR_00922] Moulder J. E., Rockwell S. (1984). Hypoxic fractions of solid tumors: experimental techniques, methods of analysis, and a survey of existing data.. Int J Radiat Oncol Biol Phys.

[OCR_00926] Olive P. L., Chaplin D. J., Durand R. E. (1985). Pharmacokinetics, binding and distribution of Hoechst 33342 in spheroids and murine tumours.. Br J Cancer.

[OCR_00931] Overgaard J., Hansen H. S., Jørgensen K., Hjelm Hansen M. (1986). Primary radiotherapy of larynx and pharynx carcinoma--an analysis of some factors influencing local control and survival.. Int J Radiat Oncol Biol Phys.

[OCR_00937] Siemann D. W., Keng P. C. (1988). Characterization of radiation resistant hypoxic cell subpopulations in KHT sarcomas. (II). Cell sorting.. Br J Cancer.

[OCR_00947] THOMLINSON R. H., GRAY L. H. (1955). The histological structure of some human lung cancers and the possible implications for radiotherapy.. Br J Cancer.

[OCR_00942] Tannock I. F. (1968). The relation between cell proliferation and the vascular system in a transplanted mouse mammary tumour.. Br J Cancer.

[OCR_00952] Teicher B. A., Lazo J. S., Sartorelli A. C. (1981). Classification of antineoplastic agents by their selective toxicities toward oxygenated and hypoxic tumor cells.. Cancer Res.

[OCR_00957] Urtasun R. C., Merz T. (1972). In vivo studies of x-irradiation damage and repair in mammalian tumor cells.. Radiology.

[OCR_00978] Watson E. R., Halnan K. E., Dische S., Saunders M. I., Cade I. S., McEwen J. B., Wiernik G., Perrins D. J., Sutherland I. (1978). Hyperbaric oxygen and radiotherapy: a Medical Research Council trial in carcinoma of the cervix.. Br J Radiol.

